# Applying Single-Cell Technology in Uveal Melanomas: Current Trends and Perspectives for Improving Uveal Melanoma Metastasis Surveillance and Tumor Profiling

**DOI:** 10.3389/fmolb.2020.611584

**Published:** 2021-01-06

**Authors:** Mona Meng Wang, Chuanfei Chen, Myoe Naing Lynn, Carlos R. Figueiredo, Wei Jian Tan, Tong Seng Lim, Sarah E. Coupland, Anita Sook Yee Chan

**Affiliations:** ^1^Singapore National Eye Centre and Singapore Eye Research Institute, Singapore, Singapore; ^2^Cytogenetics Laboratory, Department of Molecular Pathology, Singapore General Hospital, Singapore, Singapore; ^3^MediCity Research Laboratory and Institute of Biomedicine, University of Turku, Turku, Finland; ^4^A. Menarini Biomarkers Singapore Pte Ltd, Singapore, Singapore; ^5^Department of Molecular and Clinical Cancer Medicine, ITM, University of Liverpool, Liverpool, United Kingdom; ^6^Liverpool Clinical Laboratories, Royal Liverpool University Hospital, Liverpool, United Kingdom; ^7^Duke-Nus Medical School, Singapore, Singapore

**Keywords:** uveal melanoma, single-cell analysis, DEPArray NxT technology, CellSearch, circulating tumor cells, FFPE, melanoma prognostication, melanoma surveillance

## Abstract

Uveal melanoma (UM) is the most common primary adult intraocular malignancy. This rare but devastating cancer causes vision loss and confers a poor survival rate due to distant metastases. Identifying clinical and molecular features that portend a metastatic risk is an important part of UM workup and prognostication. Current UM prognostication tools are based on determining the tumor size, gene expression profile, and chromosomal rearrangements. Although we can predict the risk of metastasis fairly accurately, we cannot obtain preclinical evidence of metastasis or identify biomarkers that might form the basis of targeted therapy. These gaps in UM research might be addressed by single-cell research. Indeed, single-cell technologies are being increasingly used to identify circulating tumor cells and profile transcriptomic signatures in single, drug-resistant tumor cells. Such advances have led to the identification of suitable biomarkers for targeted treatment. Here, we review the approaches used in cutaneous melanomas and other cancers to isolate single cells and profile them at the transcriptomic and/or genomic level. We discuss how these approaches might enhance our current approach to UM management and review the emerging data from single-cell analyses in UM.

## Introduction

Uveal melanoma (UM) is the most common primary adult intraocular malignancy (Carvajal et al., 2017; Dogrusoz et al., 2017; Kaliki and Shields, 2017). Although rarely compared with other cancers, UM is a devastating disease that causes both vision loss and confers a high death rate due to metastasis that affects up to 50% of patients with primary UM (Kujala et al., 2003; Finger et al., 2005). UM metastases occur most frequently in the liver and have a poor prognosis due to limited treatment options (Eskelin et al., 2000; Buder et al., 2013; Shields and Shields, 2015). UM with a high risk of metastasis is associated with losses to chromosomes 1p, 3, 6q, 8p, a gain of 8q (Damato et al., 2007, 2011; Harbour et al., 2010; Martin et al., 2013; Field et al., 2018) and a Class 2 gene expression profile (Class 2 GEP) (Harbour and Chen, 2013; Harbour, 2014). Several somatic mutations in UM with prognostic significance have also been identified, including mutations in *BAP1, SF3B1, EIF1AX*, and *SRSF2* (Harbour et al., 2010; Martin et al., 2013; Robertson et al., 2017; Field et al., 2018). Consequently, and with the advent of new diagnostic technologies, bespoke panels for UM harnessing next-generation sequencing (NGS) to analyze both copy number variations (CNVs) and the mutational status of the genes mentioned earlier have been developed for prognostication purposes (Robertson et al., 2017; Smit et al., 2018; Thornton et al., 2020). In addition, clinicopathological features of UM—including the site and size of the primary tumor, the presence of epithelioid cells, the mitotic count, and the presence of connective tissue loops (Force, 2015)—form part of the TNM Classification of Malignant Tumors used for UM staging by oncologists to predict survival. Prognostic testing for improving metastasis surveillance and predicting survival is a concept that was first introduced by the Liverpool Ocular Oncology Centre, which continues to provide a UM surveillance service nationally and internationally (Damato et al., 2007, 2011). The Liverpool Ocular Oncology Centre has combined these parameters mentioned earlier to produce a robust prognostic algorithm that can create individualized prognostic curves for UM patients, stratifying them into groups according to metastatic risk to facilitate surveillance management (Damato et al., 2011; Cunha Rola et al., 2020).

Unfortunately, the prognostic tools currently available only allow us to identify high-risk patients requiring increased surveillance. Current therapies for metastatic UM (mUM) are still lacking and represent a notable gap in UM management. Emerging evidence suggests that analyses of circulating tumor cells (CTCs) and circulating free DNA (Anand et al., 2019) might improve mUM surveillance, particularly in high-risk UM patients. Indeed, single-cell analyses of the tumor cell microenvironment and CTCs have already provided new insights into UM tumor biology (Torres et al., 2011; Durante et al., 2020; Karlsson et al., 2020). Herein, we provide an overview of the current single-cell approaches that have transformed the field of oncology, focusing on their application in cutaneous and UMs. We also discuss novel single-cell approaches using formalin-fixed paraffin-embedded (FFPE) tissues that have potential application to UM research.

## Single-Cell Technologies in Cancer

Continual advancements in single-cell technologies have meant that their application to and utility for cancer monitoring is rapidly evolving (Navin, 2015). Technologies facilitating high-throughput single- and rare-cell isolation platforms (Wang and Navin, 2015; Valihrach et al., 2018) and single-cell NGS of DNA, RNA, proteins, and metabolites (Wang and Navin, 2015; Wills and Mead, 2015; Gawad et al., 2016; Suva and Tirosh, 2019; Lim et al., 2020) have greatly contributed to the field of oncological theragnostics.

### Single-Cell Isolation Techniques

Single-cell isolation can be used to select a homogenous tumor cell population from the surrounding, infiltrating immune cells, normal cells, or a rare cell population that comprises <1% of the total cell population (Wang and Navin, 2015; Wills and Mead, 2015; Gawad et al., 2016; Valihrach et al., 2018; Suva and Tirosh, 2019; Lim et al., 2020). Although flow cytometry, microfluidics platforms, and manual micromanipulation techniques can be used to isolate an abundant tumor-cell population, detecting, and isolating rare cancer cells are much more challenging (Wang and Navin, 2015; Valihrach et al., 2018). However, numerous methods and platforms for single-cell isolation are currently available (Table 1). Single-cell isolation techniques are particularly useful for detecting CTCs. Furthermore, interest in CTCs has been increasing, as liquid biopsies (such as blood samples) are readily accessible, and a high CTC count in such samples seems to correlate with an increased risk of metastasis in breast and other cancers (Paget, 1989; Alix-Panabières and Pantel, 2016).

**Table 1:** Methods and platforms for single-cell isolation.

**Technology**	**Platforms/methods**	**Principle of cell detection**	**Main downstream applications**
Traditional Methods	Laser-capture microdissection	Manual dissection using a laser and under microscopic visualization; requires tissue sections to be mounted on specialized slides	•Isolation of rare cells and population collection •Cell-to-cell interaction analysis
	Nanofilters	Select and sort single cells by size difference	
Commercial Platforms (low throughput, <100 cells)	CellRaft AIR System (CellMicrosystems)	Automated platform with an integrated fluorescent microscope to image, sort, and isolate cell	•Isolation of rare single cell •scRNA-Seq •scDNA-Seq •Cell phenotypes analysis •Cell clonality analysis
	AVISO CellCelector (ALS)	Fully automated image-based cell sorter that uses a robotic capillary micromanipulator to isolate cells	
	DEPArray NxT (Menarini Silicon Biosystems)	Fully automated, image-based, digital cell-isolation machine that uses antibodies and microchip technology to capture cells in dielectrophoretic cages	
	Puncher Platform (Vycap)	Automated image-based system that uses silicon chips with microwells and punching technology to isolate single cells	
Commercial Platforms (medium throughput, 100–1,000 cells)	ICELL8 Single-Cell System (Takara)	Open-platform, automated system with a built-in imaging station and software analysis tool; cells are isolated in barcoded nanowells; RNAseq reagents can be dispensed from coded chips	•scRNA-Seq
	C1 System and Polaris (Fluidigm)	Automated microfluidic system where cell capture, lysis, reverse transcription, and cell multiplexing occur in an integrated fluidic circuit chip	•scRNA-Seq •scDNA-Seq
Commercial Platforms (high throughput, more than 10,000 cells)	Chromium System (10x Genomics)	Automated, high throughput systems that use droplet-based microfluidics for integrated cell isolation and downstream analysis	•scRNA-Seq •scDNA-Seq
	Nadia (Dolomite Bio)		
	InDrop System (1CellBio)		
	ddSEQ Single-Cell Isolator (Illumina Bio-Rad)		
	Tapestri Platform (MissionBio)		
	BD Rhapsody Single-Cell Analysis System (BD)	Automated targeted transcriptomic approach using nanowells to capture single cells in combination with barcoded antibodies (Ab-seq)	•Targeted scRNA-Seq
High throughput processing specifically for CTCs	CellSearch (Menarini Silicon Biosystems)	Uses ferrofluid nanoparticles with EpCAM antibodies that detect epithelial cell adhesion molecules to magnetically separate isolated cell	•CTCs detection and isolation
	Magsweeper (Illumina Inc.)	An immunomagnetic cell separator that captures single cells with rotating magnetic rods	

A major challenge in detecting CTCs lies in their rarity in blood. As such, enrichment protocols such as ficoll density gradient separation, red blood cell lysis (erythrolysis) isolation, and immunomagnetic selection systems by Dynabeads or Miltenyi CD45 beads (Kallergi et al., 2016) have been developed and can be combined with the other single-cell isolation techniques to aid detection. Some of these single-cell isolation platforms (such as CellSearch and Magsweeper) are specific for CTC identification, as they have incorporated cytokeratin markers within their systems (Table 1) to improve CTC detection. To date, CellSearch is the only Food and Drug Administration-approved system for clinical use in breast, colorectal, and prostate CTC detection (Millner et al., 2013; De Luca et al., 2016; Kondo et al., 2017; Paolillo et al., 2017).

### Single-Cell Transcriptomics and Genomics

Single-cell transcriptomics and genomics can be used to interrogate the profile of isolated single cells of interest. Single-cell transcriptomics or single-cell RNA sequencing (scRNA-seq) (Wang and Navin, 2015; Wills and Mead, 2015; Gawad et al., 2016; Suva and Tirosh, 2019; Lim et al., 2020) provides a high-resolution transcriptomic profile of every single cell that can be used to understand the cellular function of each cell and how it interacts with other cells (Wills and Mead, 2015; Gawad et al., 2016; Suva and Tirosh, 2019). Such data have been used to define the tumor microenvironment (TME) and determine the profile of tumor-infiltrating immune cells that may confer tumor survival in breast and pancreatic carcinomas (McGranahan and Swanton, 2015; Chung et al., 2017; Davis et al., 2017; Bernard et al., 2019), thus advancing our knowledge on how such cancers behave and develop resistance.

Whole-genome amplification provides sufficient material from a single cell for single-cell genomic analysis by single-cell DNA sequencing (scDNA-seq) and array-based CNV analysis (Knouse et al., 2017; Lawson et al., 2018; Tan et al., 2019). Such approaches have been used to determine the mutational profile of resistant subclones in acute lymphoblastic leukemia, as well as colon and breast carcinomas to improve prognostication (Gawad et al., 2014; Yu et al., 2014).

## Single-Cell Approaches in Cutaneous Melanoma

As cutaneous melanomas are more common than UM, the single-cell technology is more established in cutaneous melanoma research (Joshi et al., 2014; Tirosh et al., 2016; Gerber et al., 2017). Indeed, researchers have already been able to identify and profile malignant melanoma cell clones/subclones and CTC transcriptomic signatures (Joshi et al., 2014; Tirosh et al., 2016; Gerber et al., 2017).

ScRNA-seq remains the most common approach to interrogate the molecular profiles of melanoma cells. A comprehensive scRNA-seq analysis of the benign nevus cells compared with melanoma malignant cells was used to elucidate the molecular mechanisms of early melanoma development (Kunz et al., 2018). Researchers have also combined pseudotime and scRNA-seq analyses in short-term melanoma cultures comprising cells at different stages of malignancy to detect specific gene expression changes. The findings support that a unique molecular signature underlies cutaneous melanoma transformation; this signature might represent targets for preventing disease progression (Loeffler-Wirth et al., 2018).

ScRNA-seq data have also been used to stratify melanoma cells from patients with different melanoma subtypes. In addition, scRNA-seq data have identified gene signatures for melanoma subtype classification/stratification that might be useful for improving cutaneous melanoma diagnosis and management (Gerber et al., 2017).

Treatment of metastatic melanomas was limited before the advent of BRAF inhibitors and immunotherapy. Studies of intratumor heterogeneity and the effects of such heterogeneity on melanoma resistance spurred the development of the targeted therapies and immunotherapeutics used today (Luke et al., 2017). Improving our understanding of tumor heterogeneity by performing genome-wide transcriptomics combined with single-cell phenotyping and single-cell functional proteomics will no doubt help us to understand the transition from drug-sensitive to drug-resistance cells and to identify specific signaling networks underlying melanoma drug-induced resistance (Su et al., 2017). Single-cell transcriptomic data have helped us identify several pathways associated with cellular adaptations to *BRAF* inhibitors (Luke et al., 2017). In addition, Fluidigm C1 and 10× Genomics platforms are being used to identify rare drug-resistant melanoma cellular populations; here, scRNA-seq And Klustering Evaluation has helped us to understand the response to BRAF inhibitor treatment and identify resistant signatures (Ho et al., 2018).

Single-cell isolation in the context of metastatic melanoma has also been used to study CTCs and identify antibodies to enrich for melanoma CTCs (Ulmer et al., 2004; Karakousis et al., 2013) that can be applied to UM. These studies have shown that a higher melanoma CTC count in patients correlates with shorter survival rates, metastatic tumors, and higher numbers of proliferating cells (Ulmer et al., 2004; Karakousis et al., 2013; De Souza et al., 2017).

## Current Trends in Single-Cell Technology for Uveal Melanoma

The TME of UM comprises the melanoma cells, infiltrating immune cells such as macrophages and lymphocytes, as well as supporting stromal cells and blood vessels. Initiating mutations in UM, such as *GNA1* or *GNA11*, have been extensively studied in UM. Other driver mutations include *EIF1AX* and *SF3B1*, which are associated with good and intermediate prognoses, respectively, whereas *BAP1* mutations portend a poor prognosis and the risk of metastatic disease (Ewens et al., 2014; Koopmans et al., 2014; Decatur et al., 2016; Yavuzyigitoglu et al., 2017). The loss of nuclear *BAP1* expression is associated with monosomy 3, a key alteration in clinical prognostication in UM (Farquhar et al., 2018; Smit et al., 2018; Coupland et al., 2020; Figueiredo et al., 2020; Zhang et al., 2020). Another multicentered validated prognostic test, the DecisionDx-UM™ is available in the USA and Canada and uses GEP to distinguish Class 1 UM with a low risk of metastasis and Class 2 UM with a high risk of metastasis (Harbour, 2014).

Although attempts have been made to improve our molecular understanding of the tumor microenvironment of UM by the microdissection of these subpopulations of cells and the application of NGS, these studies are still limited to the use of the bulk cells approach (Karlsson et al., 2020; Krishna et al., 2020).

Recently, Karlson et al. provided the largest whole-genome analysis of UM. In their study, they profiled mUM as well as their tumor-infiltrating lymphocytes. Compatible with previous publications (Ewens et al., 2014; Koopmans et al., 2014; Decatur et al., 2016; Yavuzyigitoglu et al., 2017), they found that *BAP1* mutations are key events in UM metastasis. However, they also noted other mutations involving *PBRM1* and *EZH2* as well as more novel mutations such as *TET1, TET2*, and *ASXL2*, which are epigenetic regulators, which occur later in metastatic development. They also detected deletions of *CDKN2a* that were associated more with metastatic tumors rather than primary UM. In profiling tumor-infiltrating lymphocytes, they found expression of PD-1, Tim3, and LAG3, which are checkpoint receptors. These findings were in agreement with data published by Figueiredo et al. (2020).

Krishna et al. (2020) also focused on interrogating the TME in mUM liver deposits. They performed NanoString-based transcriptomic profiling of 40 mUM liver samples in comparison with 6 control liver specimens. They confirmed their preliminary data (Krishna et al., 2017; Figueiredo et al., 2020) by demonstrating that the M2 macrophage actively contributed to the immunosuppressive environment within the mUM and was characterized by the upregulation of *AXNA1, CD74, CXCR4, MIF, STAT3, PLA2G6*, and *TGFB1* in addition to previously described genes, such as *LGALS3* and *HLA-DRA* (Krishna et al., 2020). This study also revealed several novel genes that were upregulated by mUM, such as *DUSP4, IRF4/MUM1*, and *CD44* (Krishna et al., 2020).

As the single-cell approaches in UM are only just emerging, the literature base is relatively sparse. Most studies have focused predominantly on CTCs in UM (see in more detail later in 4.2) (Tobal et al., 1993; Foss et al., 1995; Keilholz et al., 2004; Boldin et al., 2005; Callejo et al., 2007; Schuster et al., 2007; Fernandes et al., 2008; Ulmer et al., 2008; Pinzani et al., 2010; Suesskind et al., 2011; Torres et al., 2011; Bidard et al., 2014; Mazzini et al., 2014; Tura et al., 2014, 2016; Bande et al., 2015; Eide et al., 2015; Terai et al., 2015; Alix-Panabières and Pantel, 2016; Anand et al., 2019; Jin and Burnier, 2020).

Papers describing true single-cell approaches in UM are limited, and these include a recent paper (Durante et al., 2020) and a meeting abstract (Rodriguez et al., 2020).

### Single-Cell Approaches to Interrogate the Uveal Melanoma Microenvironment

Recently, Durante et al. (2020) have interrogated the UM TME using the single-cell approach. In their study, 59,915 tumor- and non-neoplastic single cells were isolated from eight primary and three metastatic samples and analyzed by scRNA-seq on the 10× Genomics platform. The gene expression data clustered similarly with the Class 1 and Class 2 GEP clinical prognostic test for UM. However, using single-cell resolution, of the 12 genes within these tests, *EIF1B, HTR2B, ECM1, CDH1*, and *ROBO1* genes were found to be expressed predominantly in tumor cells, whereas *SATB1*wa*s* expressed in predominantly T cells. The remaining six genes were expressed by both tumor and immune cells.

Single-cell CNV analysis revealed new evidence that canonical CNVs did not always occur as a single early event (as previously suggested) (Arozarena and Wellbrock, 2019). Rather, it seems that UM cells continue to evolve alongside tumor progression. Class 1 UMs were seen to contain loss of 1p, 3, and 8p, whereas Class 2 UMs were detected to have a gain of 6p and 6q. In addition, five tumors' initial gain of 8q followed by 8p were detected. On top of that, this result confirmed the association between signature driver mutations and canonical/non-canonical CNV subclones, which contribute to tumor progression.

More importantly, this study showed that tumor-infiltrating immune cells express the checkpoint marker LAG3 rather than PD1 or CTLA4. LAG3 might, therefore, constitute a novel candidate for immune checkpoint blockade in patients with high-risk UM, who typically exhibit a poor response to PD1 and CTLA4 checkpoint inhibition (Jindal, 2018). This approach (Durante et al., 2020) is an excellent example of how single-cell analysis can provide a higher resolution of transcriptomic changes in individual UM and tumor-infiltrating immune cells and help validate findings from bulk cell approaches (Krishna et al., 2017).

In a recent abstract publication by Rodriguez et al. (2020), they identified the novel long non-coding RNA (lncRNA) expression in UM and their association with BAP1 functionality. One hundred and three UM samples were analyzed by RNA-seq, which included quality control, trimming, alignment, and transcript discovery. From this, 104 novel transcripts were detected, of which 32 were differentially expressed between Class 1 and Class 2 UMs (Rodriguez et al., 2020). Single-cell RNA sequencing was then used to assess the tumor-specific lncRNA expression in eight primary and three mUM samples and revealed 10 lead lncRNAs to be expressed only in the tumor cells. Although further work is required, this novel data reveals the further potentials of single-cell approaches for the interrogation of UM.

### Single-Cell Approaches to Interrogate Circulating Tumor Cells in Uveal Melanoma

For detecting CTCs, some of the previous melanoma CTCs markers used include melanoma-associated chondroitin sulfate proteoglycan (Ulmer et al., 2008; Suesskind et al., 2011; Eide et al., 2015), melanoma cell adhesion molecule (CD146), NKI/beteb and NKI/C3 (Tura et al., 2014), and high-molecular-weight melanoma-associated antigen (MHW-MAA). Additionally, combining two antibodies is more efficient in isolating UM CTCs (Tura et al., 2014). However, CellSearch CTC studies, the standardized CellSearch® technique, consist of the first immunomagnetically enriching CD146 melanoma cells and subsequently staining the cell mixture with MHW-MAA. Only cells with the MHW-MAA staining and lacking CD45 and CD34 staining for leukocytes and endothelial cells, respectively, are counted as CTCs (Angi et al., 2013; Bidard et al., 2014; Bande et al., 2015; Terai et al., 2015; Anand et al., 2019).

Similar to cutaneous melanomas, data from most UM studies have supported that the CTC count in UM correlates with the extent of hepatic metastasis and thus survival (Keilholz et al., 2004; Ulmer et al., 2008; Bidard et al., 2014; Mazzini et al., 2014; Tura et al., 2016; Anand et al., 2019). However, data from a few studies performed on CellSearch contradict this and suggest no correlation with clinical parameters or tumor burden in the liver (Bande et al., 2015; Terai et al., 2015). Although the reason for this is unclear, it may be due to the increased sensitivity of CellSearch in detecting CTCs. As the clinical significance for CTCs remains unknown, single-cell analyses on CTCs detected in patients with nevi (Bande et al., 2015) and non-metastatic UM compared with CTCs present in patients with metastasis might allow us to determine their clinical significance.

One of the earliest CTC analyses in UM was conducted by Angi et al. (2013). They used the CellSearch system to detect CTCs in patients with high- and low-risk UM and showed a strong correlation between CTC presence and monosomy 3. Bande et al. (2015) also evaluated the presence of CTCs in benign choroidal nevus (premalignant), non-metastatic UM, and a case of UM with high-risk features (i.e., extrascleral extension and epithelioid histology). They found no CTCs in the benign nevi but did identify CTCs in up to 50% non-metastatic UM (<1 CTC per 7.5 ml of blood). An even higher number of CTCs were detected (3 CTCs per 7.5 ml of blood) in the case of high-risk UM. Anand et al. (2019) determined the detection frequency of CTCs in the blood in a cohort of 40 patients with primary or mUM. They confirmed that CTCs are more common in early-stage UM patients with adverse prognostic factors (Class 2 GEP and monosomy 3) and that the presence of CTCs predicts the risk of distant metastasis and poor clinical outcomes.

Others have used real-time PCR to show that the expression of biomarkers, including tyrosinase messenger RNA and MelanA/MART1 in blood, correlates with the presence of CTCs (Keilholz et al., 2004; Callejo et al., 2007; Schuster et al., 2007; Pinzani et al., 2010). With single-cell isolation and analytic techniques available, we can now determine both the single-cell expression of these and other novel biomarkers and the levels of expression that might be of metastatic relevance.

Tissue and liquid biopsy samples have been reported to show differences in molecular profiles in most cancers (Neumann et al., 2018). Intratumoral and/or spatial heterogeneity within a tumor nodule is one of the causes of such differences. Although tissue biopsies are still undertaken for diagnosis and prognostication, it is recognized that they can be limited by intratumoral heterogeneity and may not necessarily reflect the complex profile within the entire tumor. Interestingly, primary UMs suffer less from such intratumoral heterogeneity with respect to CNV (Coupland et al., 2015), although there is limited information addressing their mutational variation. Tumor biopsies may also fail to reveal the secondary molecular driver mutations that drive tumor transformation or metastasis. Liquid biopsies that include the analysis of CTC, circulating tumor DNA, and circulating microRNA have been shown to carry such information that may allow us to understand the CTC genome as they progress according to the disease status. Current studies for the detection of CTCs seem inconsistent and focus on the number of CTCs, whereas circulating tumor DNA has been shown to provide added insight with relatively good sensitivity and specificity using ultrasensitive droplet polymerase chain reactions technology (Jin and Burnier, 2020). However, this may change with the application of scRNA and scDNA on CTCs, and this may represent the future applications of single-cell technology in liquid biopsies.

### Potential Single-Cell Applications in Uveal Melanoma

**Figure 1:**
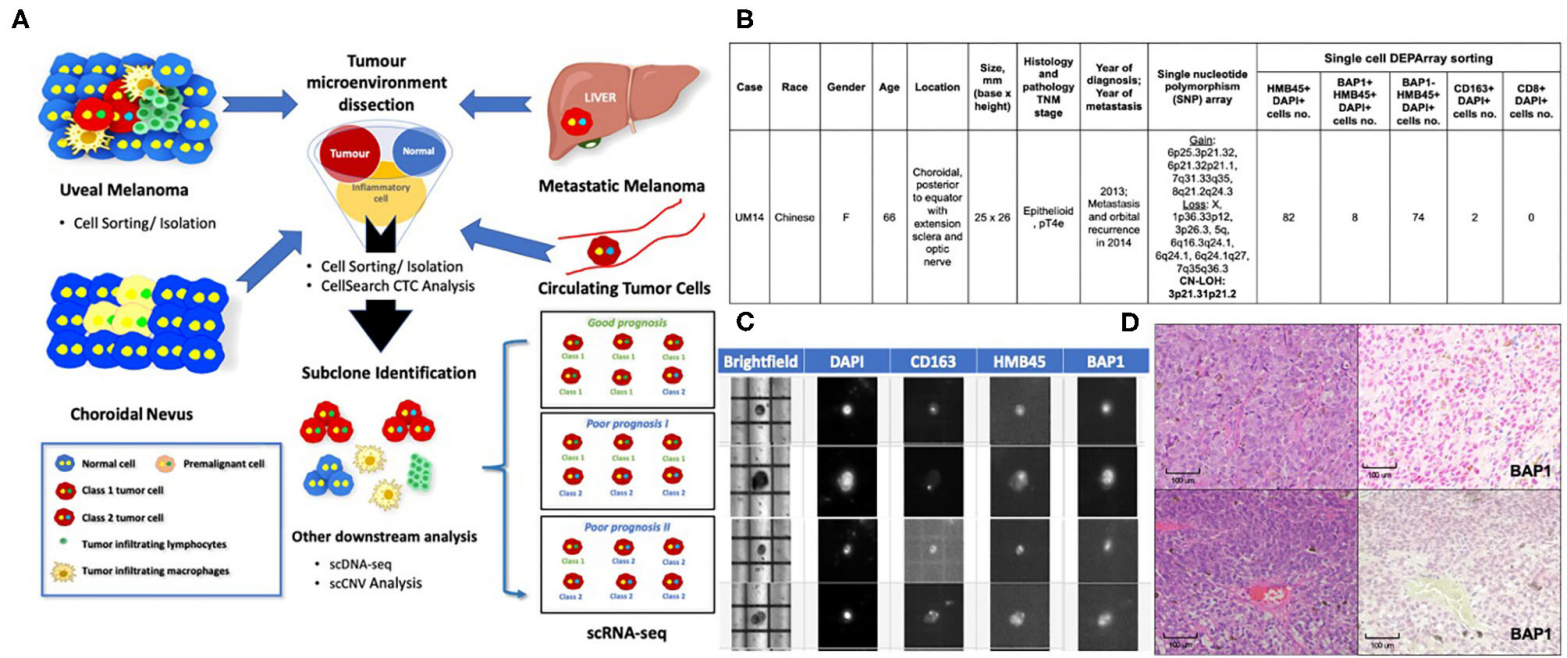
Applications of Single-Cell Technology in Uveal Melanoma. **(A)** Potential applications of single-cell analysis in UM. *In premalignant choroidal nevi:* Single-cell analysis can be used to study the mechanisms that underlie malignant transformation and identify biomarkers for early detection and even prevention. *In uveal melanomas:* Single-cell analysis can be used to study the tumor microenvironment to determine how immune cells and stromal cells might contribute to tumor-cell survival. Similar applications in treated and untreated melanoma cells or patient samples resistant to treatment might allow us to identify subclones of malignant cells and subsequently develop personalized or targeted therapies for drug-resistant UM. *In the blood:* Studying the nature of CTCs will allow us to determine which CTCs are likely to result in metastatic deposits. *In the liver and other organs:* Studying metastatic cells vs. primary UM cells might allow us to identify biomarkers and the underlying mechanisms of metastatic disease, which will allow us to develop treatment interventions. Single-cell Workflow: Tumor microenvironment is dissected using single-cell sorting tools. Subclone populations may then be identified and downstream analysis performed on each individual cell. Currently, scRNA-seq is the most commonly used approach to interrogate the TME and has been used to confirm bulk cell gene expression analysis of Class 1 and Class 2 UMs at a single cell level (Durante et al.). Other techniques that are being explored include scDNA-seq and scCNV analyses. **(B)** DEParray NXT™ analysis demonstrating the detection of BAP1-negative and BAP1-positive cells with activated CD163 macrophages in a patient sample that showed high-risk histology features of epithelioid histology and large basal diameter and height. The patient also had copy neutral-loss of heterozygosity on 3p21.31p21.2 and gain of 8q, losses of 1p and 6q that are recognized as high-risk cytogenetic features. **(C)** DEParray NXT™ brightfield image demonstrating how single cells can be verified and debris and duplets can be excluded for a higher quality of sampling. **(D)** Histology from patient sample UM14 that had high-risk cytogenetic features. Top panel, Left panel: Hematoxylin and eosin stain showing the epithelioid cytology. Right panel: BAP1 immunohistochemistry staining shows *no* loss of BAP1 expression. Lower panel, Left panel: Hematoxylin and eosin stain showing the epithelioid cytology. Right panel: BAP1 IHC staining show *loss* of BAP1 expression.

Based on the applications in cutaneous melanomas and other cancers, we propose a customized single-cell workflow for UM research (Figure 1A). First, single-cell genomic profiling by scRNA-seq and/or scDNA-seq can be used to elucidate the molecular events underlying the transformation of a premalignant nevus to invasive UM with metastasis. In addition, we can identify tumor heterogeneity and the temporal and spatial evolution of tumor subclones to help predict resistant cell populations. Combining single-cell isolation techniques with scRNA-seq and scDNA-seq, circulating single melanoma cells in the blood may be identified, profiled and compared with the primary UM clone to determine the metastatic potential of CTCs.

### Single-Cell Technology Using Formalin-Fixed Paraffin-Embedded Uveal Melanoma Tissues

As UM is a rare disease, fresh tissue specimens are not readily available for research use; as such, many rely on melanoma cell lines (Gerber et al., 2017). In Asia, where UM has a particularly low incidence (0.3–0.4 per million per year vs. 5–8 million per year in Western countries) (Kaliki and Shields, 2017; Tan et al., 2020), FFPE archival UM tissues are readily available. We previously established the DEPArray NxT workflow for intraocular lymphoma single-cell isolation, followed by clonality and genomic analyses (Tan et al., 2019). Using this approach, we provide proof-of-concept that other single-cell sorting techniques, other than 10× genomics (used by Durante et al.), may also be utilized for dissecting the UM microenvironment. Using a modified single-cell isolation protocol for FFPE tissues, we isolated UM cells and infiltrating immune cells by selecting specific cell populations by antibody staining image analysis and visual selection using the DEPArray Nxt system (Figures 1B,C). We compared the findings with current clinical prognostic standards that included histology, BAP1 immunohistochemistry, and Affymetrix Chromosomal MicroArray analysis to demonstrate that it is possible to detect single cells with DEPArray Nxt with the immunophenotype corresponding to the slide samples.

Using this DEPArray single-cell image-screening approach (Figure 1C), it was possible to isolate and quantify single UM cells with or without BAP1 nuclear expression and immune cells, such as CD8 T cells and CD163+ activated macrophages, from archival UM FFPE samples (Figure 1B). We showed (Figures 1B–D) that we were able to correlate the loss of nuclear BAP1 expression (Figure 1D) in individual UM cells from a patient who presented with a large posterior choroid melanoma with epithelioid histology (Figure 1D) and advanced TNM staging (Figure 1B). Single nucleotide polymorphism array detected copy neutral loss of heterozygosity on 3p21.31p21.2 and gain of 8q, losses of 1p and 6q (Figure 1B). Of note, copy neutral loss of heterozygosity of chromosome 3 is superior to monosomy 3 in predicting metastasis (Onken et al., 2004). The loss of nuclear BAP1 (detected on our sample by immunohistochemistry) is also associated with an immunosuppressive microenvironment in UM patients (Figueiredo et al., 2020), which aligns with the low level of activated CD163 macrophages and absence of CD8 T cells in the histology of this UM sample (Figure 1B). This single-cell approach may be used to isolate specific cells from the UM microenvironment for further downstream, single-cell transcriptomic and genomic analyses.

Although the genomic quality of this single-cell approach has not been validated, it is a proof-of-concept demonstration that such microdissection can be performed in UM, especially when there is a rare cell population that cannot be detected by routine flow cytometry.

Despite the difficulties in performing scRNA-seq, scDNA-seq, and scCNV analyses on FFPE tissues, due to nucleic acid alterations after formalin fixation, recent studies have optimized such protocols to allow single-cell transcriptomic (Foley et al., 2019) and genomics (Martelotto et al., 2017) in FFPE specimens to achieve results comparable with frozen or fresh samples (Hedegaard et al., 2014).

## Discussion

Applying single-cell technology to UM has largely been restricted to identifying CTCs and limited to making correlations with clinical prognostic features. However, current single-cell technologies allow for transcriptomic and genomic analyses to be performed on isolated CTCs. By interrogating CTCs by scRNA-seq, scDNA-seq, and sc-CNV at the early and metastatic stages of UM and comparing the data with those derived from the original UM clone and/or the hepatic metastatic clone, it may be possible to elucidate the specific signature of malignant and non-malignant CTCs. The increasing success in detecting CTCs with the CellSearch platform suggests that early surveillance of circulating melanoma cells may soon become a reality, especially as CellSearch has already been Food and Drug Administration-approved for clinical use in other cancers.

As mentioned, scRNA-seq analysis of the TME of UM has led to the discovery that UM immune cells express LAG3 rather than the traditional ligands used in immunotherapy. This finding, together with the demonstration of fibrotic collagenous bands surrounding mUM, explains why there is an immunosuppressive environment within these tumor nodules and why current immunotherapies are not successful in UM. Novel treatment strategies are, therefore, urgently required. Further interrogation of the TME in UM hepatic metastases and drug-resistant tumor cells may reveal novel targets and biomarkers for such new targeted therapies.

Small sample sizes greatly limit the study power in mUM research. Moreover, single-cell tools are still not widely available in all institutions and countries. International collaborations that combine multicenter, single-cell research are thus crucial for the development and validation of novel findings. Such international collaborations might reveal genomic and transcriptomic differences that underlie the differences in UM survival and outcomes in different countries. Finally, forming an mUM research consortium will likely help develop standardized protocols that will ensure consistency in data production and a high standard of single-cell research.

We propose that FFPE tissues can be used to overcome sample size limitations and generate pilot data before sourcing fresh tissues for validation. We have developed an FFPE single-cell approach using the DEParray platform that seems representative of the UM tumor microenvironment. Pitfalls associated with FFPE NGS analyses can be minimized with image-based single-cell isolation to review single cells before selection using brightfield or fluorescence images to eliminate false staining by debris or duplets and improve the quality of the isolated cells. Together with new techniques that improve the quality of FFPE RNA and DNA, this FFPE single-cell approach might constitute an alternative to help advance UM research.

With these opportunities, we hope that the next decade of UM research will see an increase in single-cell-related studies that will lead to the development of clinically applicable tools for metastatic surveillance and new therapies.

## Data Availability Statement

The raw data supporting the conclusions of this article will be made available by the authors, without undue reservation.

## Ethics Statement

The Centralized Institutional Review Board of SingHealth provided ethical approval for the use of patient materials in this study. The patients/participants provided their written informed consent to participate in this study. Patients data and samples were anonymized after initial collection.

## Author Contributions

AC designed the experiments and provided clinical material and data. MW, CC, and ML performed the experiments. MW and AC wrote the manuscript. All authors reviewed and approved the manuscript submission.

## Conflict of Interest

WT and TL are researchers for A. Menarini Biomarkers Singapore Pte Ltd. AC received research funding from A. Menarini Biomarkers Singapore Pte Ltd. The remaining authors declare that the research was conducted in the absence of any commercial or financial relationships that could be construed as a potential conflict of interest.
